# Pangolins Lack IFIH1/MDA5, a Cytoplasmic RNA Sensor That Initiates Innate Immune Defense Upon Coronavirus Infection

**DOI:** 10.3389/fimmu.2020.00939

**Published:** 2020-05-08

**Authors:** Heinz Fischer, Erwin Tschachler, Leopold Eckhart

**Affiliations:** ^1^Division of Cell and Developmental Biology, Center for Anatomy and Cell Biology, Medical University of Vienna, Vienna, Austria; ^2^Department of Dermatology, Medical University of Vienna, Vienna, Austria

**Keywords:** zoonosis, pangolin, coronavirus, RNA sensor, innate immunity, inflammation, tolerance, gene loss

## Abstract

Zoonotic infections are an imminent threat to human health. Pangolins were recently identified as carriers and intermediate hosts of coronaviruses. Previous research has shown that infection with coronaviruses activates an innate immune response upon sensing of viral RNA by interferon-induced with helicase C domain 1 (IFIH1), also known as MDA5. Here, we performed a comparative genomics study of RNA sensor genes in three species of pangolins. DDX58/RIG-I, a sensor of cytoplasmic viral RNA and toll-like receptors (TLR) 3, 7, and 8, which bind RNA in endosomes, are conserved in pangolins. By contrast, IFIH1 a sensor of intracellular double-stranded RNA, has been inactivated by mutations in pangolins. Likewise, Z-DNA-binding protein (ZBP1), which senses both Z-DNA and Z-RNA, has been lost during the evolution of pangolins. These results suggest that the innate immune response to viruses differs significantly between pangolins and other mammals, including humans. We put forward the hypothesis that loss of IFIH1 and ZBP1 provided an evolutionary advantage by reducing inflammation-induced damage to host tissues and thereby contributed to a switch from resistance to tolerance of viral infections in pangolins.

## Introduction

Emerging infectious diseases represent a major challenge to public health. The transmission of pathogens from other vertebrate animals to humans is of particular concern because the resulting diseases, known as zoonoses, have caused major epidemics in the past and continue to pose enormous threats to the human population, as exemplified by the recent severe acute respiratory syndrome coronavirus 2 (SARS-CoV-2) outbreak (1, 2). In a broader sense, viral and bacterial pathogens are among the strongest drivers of evolutionary change and the genomes of vertebrate species have been shaped, to a large extent, by adaptations to pathogens.

To cope with viral infections, vertebrate species have evolved response strategies which can be classified into resistance and tolerance (3). Resistance depends on the efficient sensing of the infection and mounting of antiviral responses that involve programmed death of infected cells, suppression of viral replication, inflammation and the establishment of adaptive immunity. However, pathogens can also trigger overreactions of the immune system which cause more harm to the individual than the infectious agent itself (4, 5). Therefore, tolerance to infections has evolved as an alternative response of many hosts to specific pathogens (6, 7). In this scenario, the pathogens are not efficiently eliminated but the pathogen or defense-induced damage to the host is reduced. Tolerance does not depend on, or is even impeded by, the early sensing of pathogen-associated patterns (PAMPs) and its mechanisms of protection are not yet fully understood (6, 8, 9). Species that tolerate infections can carry a high burden of infectious agents, and therefore may be important reservoirs for transmissions to other species. This notion is supported by the finding that bats tolerate many viral infections some of which have spread to humans causing zoonoses such as Ebola, severe acute respiratory syndrome (SARS) and Middle East respiratory syndrome (MERS) (7).

Pangolins have been identified, besides bats, as a possible source of severe acute respiratory syndrome coronavirus 2 (SARS-CoV-2), the cause of coronavirus disease 2019 (COVID-19) (10–14). Eight species of pangolins form the mammalian order Pholidota which is most closely related to Carnivora (cat-like and dog-like carnivorans). They are insectivorous and toothless animals whose body is largely covered by keratinous scales. The immune defense of pangolins has not been characterized yet except for reports on the deficiencies of TLR5, a receptor of bacterial flagellin (15) and interferon-ε, an antiviral cytokine of epithelia (16, 17). The receptor of SARS-CoV-2, i.e., angiotensin I converting enzyme 2 (ACE2) is conserved in pangolins (18) and coronaviruses isolated from pangolins have a receptor binding domain in their spike protein that is uniquely similar to that of SARS-CoV-2 (10, 19).

Antiviral defense of vertebrates is initiated by sensors of viral nucleic acids. Infections with RNA viruses, such as coronaviruses, influenza viruses and Ebolavirus activate sensors of extracellular or endosomal RNA, such as TLR3, TLR7, and TLR8 (20), and sensors of intracellular RNA, such as IFIH1/MDA5, ZBP1, and DDX58/RIG-I (21–28). These sensors are specific for different subtypes of RNAs that constitute the viral genome or appear during viral replication or gene expression and they activate distinct cellular and organismal responses, such as necroptotic cell death, interferon signaling and inflammation (27, 29).

Here we report a unique degeneration of the innate immune response against RNA viruses in pangolins.

## Materials and Methods

The following genome sequences of pangolin species were analyzed: Malayan pangolin (*Manis javanica*), Assembly: ManJav1.0 (GCA_001685135.1), submitted by The International Pangolin Research Consortium (16); Chinese pangolin (*Manis pentadactyla*), Assembly: M_pentadactyla-1.1.1 (GCA_000738955.1), submitted by Washington University; Tree pangolin (*Manis tricuspis*), Assembly: ManTri_v1_BIUU (GCA_004765945.1), submitted by Broad Institute. Gene annotations were available in GenBank only for *M. javanica* (NCBI Manis javanica Annotation Release 100).

Shared order of gene arrangement (synteny) in the Malayan pangolin (*M. javanica*), cat, dog, cattle, mouse, and human was assessed by comparison of gene loci that were downloaded from GenBank at https://www.ncbi.nlm.nih.gov/gene/ (last accessed on 27 March, 2020). In addition, Basic Local Alignment Search Tool (BLAST) was used to find regions of local similarity between sequences (30). Amino acid and nucleotide sequence were aligned with the Multalin software (31). Divergence times of evolutionary lineages were obtained from the Timetree website (www.timetree.org) (32).

## Results

### *IFIH1* Is a Pseudogene in Pangolins

**Figure 1:**
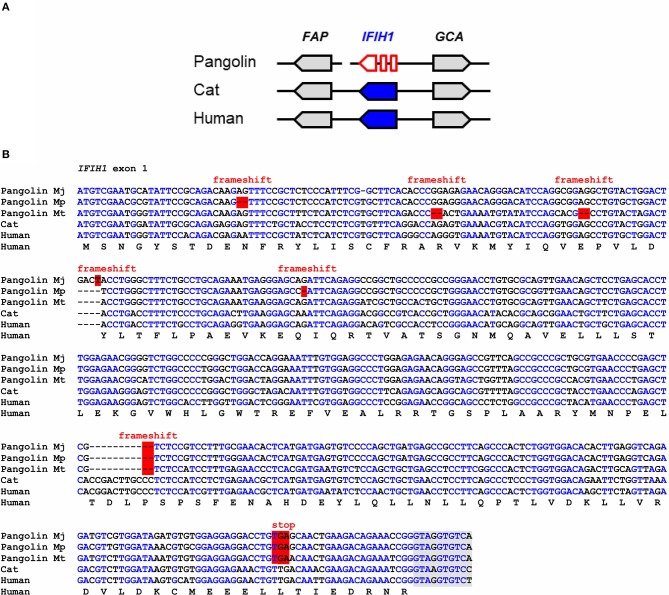
*IFIH1* is a pseudogene in pangolins. **(A)** Gene locus of *IFIH1* in the pangolin (*M. javanica*), cat, and human. Genes are represented by arrows pointing in the direction of transcription. A sequence gap is located between *FAP* and *IFIH1* in the pangolin. **(B)** Inactivating mutations in exon 1 of *IFIH1* in three species of pangolins. Nucleotide sequences of pangolins, cat and human were aligned. The coding sequence of human *IFIH1* was translated and the amino acid sequence is shown below the nucleotide sequences. Frameshift mutations and in-frame stop codons are highlighted by red shading. Nucleotides conserved in more than 50% of the sequences are indicated by blue fonts. Nucleotides in the flanking region of the first intron are shown with gray shading. Nucleotide sequence accession numbers (GenBank): Human (NC_000002.12, nucl. 162317845-162318307, compl.), cat (NC_018730.3, nucl. 154125204-154125666, compl.), Malayan pangolin (NW_016533891.1, nucl. 53417-53871, compl.), Chinese pangolin (JPTV01003556.1, nucl. 39028-39476, compl.), tree pangolin (SOZM010146646.1, nucl. 741-1188, compl.). Abbreviations: compl., complementary; nucl., nucleotide numbers; Mj, *Manis javanica*; Mp, *Manis pentadactyla*; Mt, *Manis tricuspis*.

IFIH1, also known as melanoma differentiation-associated protein 5 (MDA5), binds to double-stranded RNA in the cytosol and signals through mitochondrial antiviral-signaling protein (MAVS) to activate expression of interferons and to induce inflammation (33). IFIH1 senses cytoplasmic RNA of coronaviruses and other viruses (27, 34, 35). Comparison of the *IFIH1* gene locus showed conservation of the arrangement of *IFIH1* relative to the neighboring genes in mammals (Figure 1A). In the Malayan pangolin, *IFIH1* is inactivated by more than 10 frameshift and in-frame stop mutations. *In silico* translation of the pangolin *IFIH1* pseudogene (GenBank gene ID: 108398082) and alignment of the resulting amino acid sequence to that of human IFIH1 showed numerous disruptive mutations (Figure S1A). An open reading frame in exon 1 of the Malayan pangolin encodes a theoretical protein that lacks essential domains and has only 100 amino acid residues whereas functional IFIH1 proteins consist of more than 1,000 amino acid residues (Figure S2). Detailed comparative analysis of exon 1 showed the presence of multiple frameshift mutations and in-frame stop codons in the *IFIH1* genes of Malayan, Chinese and tree pangolins (Figure 1B). One of the frameshift mutations and one premature stop mutation are shared by all three species, suggesting that these mutations have already been present in their last common ancestor that lived more than 20 million years ago (32).

### *ZBP1* Is a Pseudogene in Pangolins

**Figure 2:**
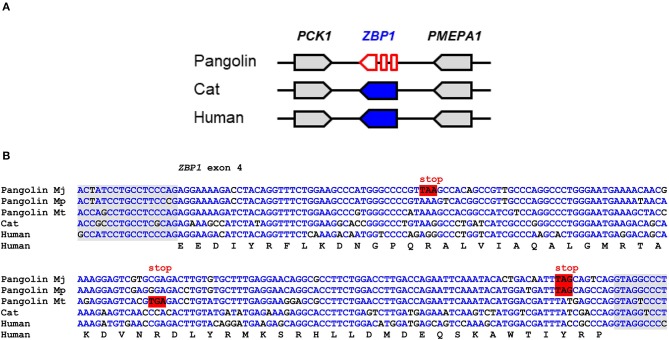
*ZBP1* is a pseudogene in pangolins. **(A)** Gene locus of *ZBP1* in the pangolin (*M. javanica*), cat, and human. Genes are represented by arrows pointing in the direction of transcription. **(B)** Inactivating mutations in exon 4 of *ZBP1* in three species of pangolins. Nucleotide sequences of pangolins, cat and human were aligned. The coding sequence of human *ZBP1* was translated and the amino acid sequence is shown below the nucleotide sequences. In-frame stop codons are highlighted by red shading. Nucleotides conserved in more than 50% of the sequences are indicated by blue fonts. Nucleotides in the flanking region of the introns are shown with gray shading. Nucleotide sequence accession numbers (GenBank): Human (NC_000020.11, nucl. 57614878.0.57615077, compl.), cat (NC_018725.3, nucl. 5721658-5721857), Malayan pangolin (NW_016529116.1, nucl. 156452-156651, compl.), Chinese pangolin (JPTV01006633.1, nucl. 23295.0.23494), tree pangolin (SOZM010101098.1, nucl. 532-731). Abbreviations: compl., complementary; nucl., nucleotide numbers; Mj, *Manis javanica*; Mp, *Manis pentadactyla*; Mt, *Manis tricuspis*.

ZBP1 binds to left-handed double helix structures of DNA and RNA (Z-DNA and Z-RNA) and thereupon triggers necroptosis and inflammation through interactions with receptor-interacting serine/threonine-protein kinase 3 (RIPK3) (36). Influenza virus and other viruses induce ZBP1-mediated innate immune responses in humans and mice (24, 25). Comparison of the *ZBP1* gene locus showed conservation of the arrangement of *ZBP1* relative to the neighboring genes in mammals (Figure 2A). In the Malayan pangolin, *ZBP1* is inactivated by multiple in-frame stop codons. *In silico* translation of the pangolin *ZBP1* pseudogene (GenBank gene ID: 108390931) and alignment of the resulting amino acid sequence to that of human ZBP1 showed premature termination of the translation product and lack of the carboxy-terminal half of the protein (Figure S1B). Mutations that prevent the production of a functional protein were found in all segments of the *ZBP1* pseudogene of the Malayan pangolin. The nucleotide sequence alignment of *ZBP1* exon 4 shows the presence of in-frame stop codons in three species of pangolins (*M. javanica, M. pentadactyla, M. tricuspis*) (Figure 2B).

In contrast to *IFIH1* and *ZBP1*, the genes encoding the intracellular RNA sensor RIG-I, i.e., *DExD/H-box helicase 58* (*DDX58*), and *TLR3, TLR7*, and *TLR8* which control the sensing of RNA in endosomes and a series of other genes involved in antiviral signaling and defense, such as *MAVS, RIPK3, MLKL, SKIV2L, OAS2, RNASEL*, and *EIF2AK2* (PKR) do not contain disruptive mutations and therefore appear to be intact in the Malayan pangolin (*M. javanica*) (Table S1). *DDX58* contains in-frame stop codons and frameshift mutations in the tree pangolin (*M. tricuspis*) but not in the Chinese pangolin (*M. pentadactyla*) (Figure S3), suggesting that the tree pangolin lacks functional DDX58/RIG-I in addition to the two intracellular RNA sensors (IFIH1 and ZBP1) absent in all pangolins.

### Pangolins Have Lost *IFIH1* and *ZBP1* After Their Evolutionary Divergence From Other Mammalian Lineages

**Figure 3:**
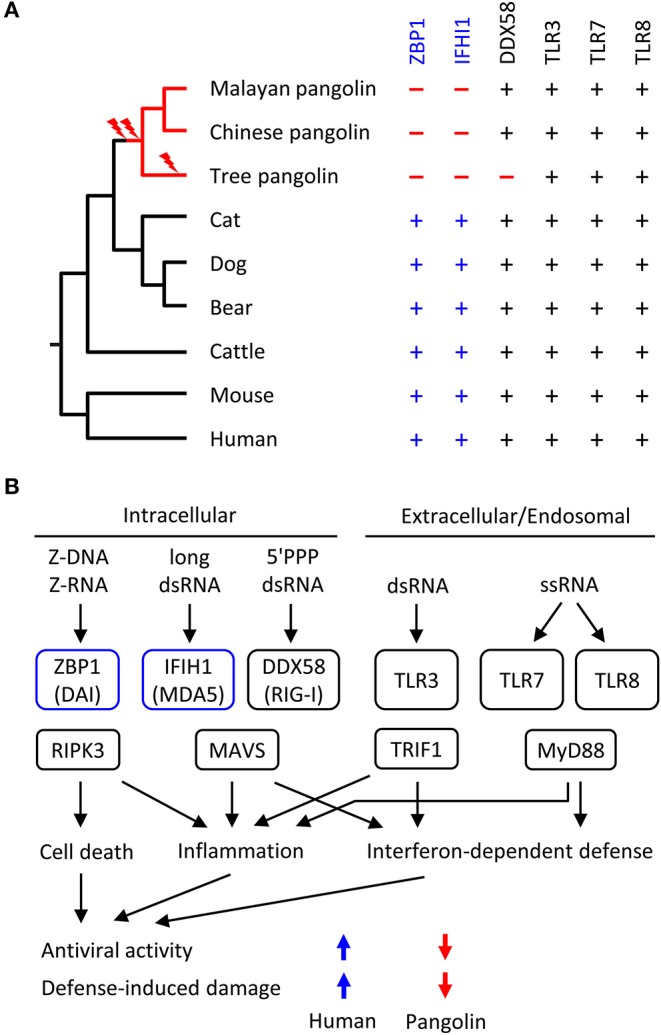
Evolution of RNA sensor genes and possible implications on antiviral responses in pangolins. **(A)** Phylogenetic tree of mammals and comparison of presence (+) or absence (–) of RNA sensor genes. Evolutionary gene loss (indicated by lightning bolt symbols) was inferred from the species distribution of the genes. Species: Malayan pangolin (*Manis javanica*), Chinese pangolin (*Manis pentadactyla*), tree pangolin (*Manis tricuspis*), cat (*Felis catus*), dog (*Canis lupus familiaris*), bear (*Ursus arctos horribilis*), cattle (*Bos taurus*), mouse (*Mus musculus*), human (*Homo sapiens*). **(B)** Schematic overview of innate immune sensors of viral RNA and signaling in mammals. Only RNA sensors investigated in this study are shown. The schematic includes the hypothesis about IFIH1 and ZBP1-dependent differences in the antiviral activity and defense-induced damage to the host. The directions of the colored arrows indicate the effects of the presence or absence of RNA sensors. 5'PPP, triphosphorylated at the 5'-end; ds, double-stranded; ss, single-stranded.

We screened the genomes of mammals from diverse phylogenetic lineages for functional copies (devoid of frameshift mutations and premature in-frame stop codons) of *ZBP1, IFIH1* and other RNA sensor genes. Mapping the presence or absence of these genes onto the phylogenetic tree suggested that loss of both *ZBP1* and *IFIH1* occurred in the pangolin lineages soon after divergence from the lineage leading to Carnivora (represented by cat, dog and bear in Figure 3A). Other genes implicated in anti-RNA-viral defense are conserved in the selected set of species (Figure 3A; Table S2).

## Discussion

Based on the known target specificities of mammalian RNA sensors (Figure 3B), the loss of *ZBP1* and *IFIH1* suggests that the response to Z-RNA and long double-stranded RNA is diminished in pangolins. Accordingly, the resistance to RNA viruses that depend on cytoplasmic Z-RNA and long double-stranded RNA for replication has likely decreased in the evolution of pangolins. We put forward the hypothesis that strong antiviral defense was harmful and loss of *ZBP1* and *IFIH1* provided an evolutionary advantage by increasing tolerance to infections by certain RNA viruses, including coronaviruses.

Viruses are potent drivers of evolutionary adaptations in their hosts. Both insufficient and overshooting responses to viral infections have deleterious effects, leading to strong selection for resistant or tolerant host genotypes (37, 38). Bats have retained functional RNA sensor genes (Table S3) but exert only dampened antiviral responses, indicating that they have adapted to the evolutionary pressure from viruses by decreasing inflammatory responses and by enhancing tolerance to viral replication (39–42). The results of the present study suggest that pangolins are another group of mammals with evolutionarily downregulated defense against a subset of viruses, namely those sensed by IFIH1/MDA5 or ZBP1 in other species. Our data urge to study the virus burden of pangolins, their antiviral immune response and their ability to act as reservoirs for viruses with zoonotic potential, especially coronaviruses. While genetic suppression of IFIH1/MDA5 and ZBP1-dependent pathways had neutral or beneficial effects in the evolution of pangolins, pharmaceutical suppression of IFIH1/MDA5 and ZBP1-dependent signaling may be beneficial for human patients with overreactions to viral nucleic acids.

## Data Availability Statement

All datasets presented in this study are included in the article/Supplementary Material.

## Author Contributions

HF and LE designed the study and performed bioinformatic analyses. HF, ET, and LE wrote the manuscript.

## Conflict of Interest

The authors declare that the research was conducted in the absence of any commercial or financial relationships that could be construed as a potential conflict of interest.
