# Supplementary material for: Pangolins Lack IFIH1/MDA5, a Cytoplasmic RNA Sensor That Initiates Innate Immune Defense Upon Coronavirus Infection
Source: Front Immunol. 2020 May 8;11:939. doi: 10.3389/fimmu.2020.00939 (PMC7225364; doi:10.3389/fimmu.2020.00939)
Supplement: Supplementary file 2 [file Image_1.pdf]

A

Amino acid sequence alignment: Translation of pangolin *IFIH1* pseudogene and human IFIH1 protein

```
          1                                                                                                  100
Pangolin  MSNAYSADKSFRSPISASHPERTGTSRRRLYWTDYLGFLPAEMREQIQRPAAPAGNLCAVEQLLSTLENGVWPPGWTRKFVEALERTGSRSAARCVNPEL
   Human  MSNGYSTDENFRYLISCFRA-RVKMYIQVEPVLDYLTFLPAEVKEQIQRTVATSGNMQAVELLLSTLEKGVWHLGWTREFVEALRRTGSPLAARYMNPEL

          101                                                                                                200
Pangolin  X---SPSFANTHDECPQLMSRLQPTLVDTLEVRDVVDRCVEEDLXATEDRNRIVAAENIGNESGIRELLKRIMEKQNWFSTFLTILHSK------RDMMG
   Human  TDLPSPSFENAHDEYLQLLNLLQPTLVDKLLVRDVLDKCMEEELLTIEDRNRIAAAENNGNESGVRELLKRIVQKENWFSAFLNVLRQTGNNELVQELTG

          201                                                                                                300
Pangolin  FKCCAVSSETENLTQ-DGPEVKESHLLAAVQPSPEKEAWDVENNSLESSFMDSYIVSESDTSLAEGSVSCLDESLGHNSNMGSDSGTAGSDSGEENMPRR
   Human  SDCSESNAEIENLSQVDGPQVEEQLLSTTVQPNLEKEVWGMENNSSESSFADSSVVSESDTSLAEGSVSCLDESLGHNSNMGSDSGTMGSDSDEENVAAR

          301                                                                                                400
Pangolin  SSPENCLSGLTKWKRPNQXELPLGPYQMEAAQPALEEKNTIICLLPTGSGKTRASGYVAKDHLDERKQPSEPGNVMVLVNKHFXVPLVKQFFCKEFKPFL
   Human  ASPEP--------------ELQLRPYQMEVAQPALEGKNIIICL-PTGSGKTRVAVYIAKDHLDKKKKASEPGKVIVLVNK---VLLVEQLFRKEFQPFL

          401                                                                                                500
Pangolin  KKWYQVTGLSVDTQLKISFPEVVKSCDVIISTAQILKNSLLNPENGEDTGVQFSDFSLIIIDECHHTKKEDVYNNVMRCYLKQKLKNNRLKEENKPVIPL
   Human  KKWYRVIGLSGDTQLKISFPEVVKSCDIIISTAQILENSLLNLENGEDAGVQLSDFSLIIIDECHHTNKEAVYNNIMRHYLMQKLKNNRLKKENKPVIPL

          501                                                                                                600
Pangolin  PQIPGLTASPGAGGATKQAQAKEHILKICANLDAFTIKTMKENIEQLKDQIKEPCKKFVISDDTREDQFKDKIIAIMTSIQSFCQVCPMPDFGTQPYEQC
   Human  PQILGLTASPGVGGATKQAKAEEHILKLCANLDAFTIKTVKENLDQLKNQIQEPCKKFAIADATREDPFKEKLLEIMTRIQTYCQMSPMSDFGTQPYEQW

          601                                                                                                700
Pangolin  GIQIEKKAAKEGNCRDRACAEHLRKPNGTLQINGTIXMIDAYRHLETFYNDEKEKS-------GDSGGN-----GDEGEGDKKKALKLDGADDFLMNSFL
   Human  AIQMEKKAAKEGNRKERVCAEHLRKYNEALQINDTIRMIDAYTHLETFYNEEKDKKFAVIEDDSDEGGDDEYCDGDEDEDDLKKPLKLDETDRFLMTLFF

          701                                                                                                800
Pangolin  DNKKTLKKLAANPEHENEKLTELRSSILGQSARTEESAXGIIFTKTRQSVYALSQRISELSTFAEVGVKAQHVTGAGHSSEFKPATQNEQKEVISKFHTG
   Human  ENNKMLKRLAENPEYENEKLTKLRNTIMEQYTRTEESARGIIFTKTRQSAYALSQWITENEKFAEVGVKAHHLIGAGHSSEFKPMTQNEQKEVISKFRTG

          801                                                                                                900
Pangolin  KIHLLIATAVAEEGLDIKECNIVSRYDLITDETALVQARAGARAEESTXVLVGHSGSGAFELATVNDFQEKMTYKAIDHVQNMKPDEYAHKILEVYMQSI
   Human  KINLLIATTVAEEGLDIKECNIVIRYGLVTNEIAMVQARGRARADESTYVLVAHSGSGVIEHETVNDFREKMMYKAIHCVQNMKPEEYAHKILELQMQSI

          901                                                                                                1000
Pangolin  MEKKIKIKRSRAKXYRDKPSLISHLCKLCSVLACPSEXYHVIERMRQGHVTAEFKELCLVRENKALIKKFAHYQANGKIICTKCGQAWGTMMVHKGLDLP
   Human  MEKKMKTKRNIAKHYKNNPSLITFLCKNCSVLACSGEDIHVIEKMHHVNMTPEFKELYIVRENKALQKKCADYQINGEIIC-KCGQAWGTMMVHKGLDLP

          1001                                        1045
Pangolin  CLKXKNFVMVFKNNKPKKQYKKXVEFPVTFPGLEYSLYCLLSDED
   Human  CLKIRNFVVVFKNNSTKKQYKKWVELPITFPNLDYSECCLFSDED
```

B

Amino acid sequence alignment: Translation of pangolin *ZBP1* pseudogene and human ZBP1 protein

```
          1                                                                                                  100
Pangolin  MAEVPADPGKEGYPEKKTLQVLRDAGSPVKTAXLAGDCQELKKKLHQVLYRLKKESKVALEGQATVAPLWGWGWSEEKTYRFLEAHGPRXATAVAQALGM
   Human  MAQAPADPGREAERPQQHAATIPETPGPQFSQQ-----------------------------------------REEDIYRFLKDNGPQRALVIAQALGM

          101                                                                                                200
Pangolin  KTTKGVVRDLCALRNRRLLDLDQNSNTLTIXQSEDSGGRNQSTTVVYQQNPINMICQKGPNSRISTENSGGIQIGHGHVTVRQTAPRE--SVLPLVLQLR
   Human  RTAKDVNRDLYRMKSRHLLDMDEQSKAWTIYRPEDSGRRAKSASIIYQHNPINMICQNGPNSWISIANSEAIQIGHGNIITRQTVSREDGSAGPRHLPSM

          201                                                                                                300
Pangolin  VPW-LDPGGTQTSTWRGVYSDRCSWDTATR----------------------------------------------------------------------
   Human  APGDSSTWGTLVDPW-GPQDIHMEQSILRRVQLGHSNEMRLHGVPSEGPAHIPPGSPPVSATAAGPEASFEARIPSPGTHPEGEAAQRIHMKSCFLEDAT

          301                                                                                            396
Pangolin  ------------------------------------------------------------------------------------------------
   Human  IGNSNKMSISPGVAGPGGVAGSGEGEPGEDAGRRPADTQSRSHFPRDIGQPITPSHSKLTPKLETMTLGNRSHKAAEGSHYVDEASHEGSWWGGGI
```

Supplementary Figure S1. Positions of disruptive mutations within the *in silico* translation of *IFIH1* and *ZBP1* pseudogenes of the Malayan pangolin. (A) The pangolin *IFIH1* pseudogene was translated whereby in-frame stop codons and frameshift mutations were marked by "X" on red background (GenBank accession number XP_017517341.1). This theoretical amino acid sequence was aligned to that of human IFIH1. Identical amino acid residues are indicated by blue fonts. Numbers indicate amino acid positions. (B) The pangolin *ZBP1* pseudogene was translated whereby in-frame stop codons and frameshift mutations were marked by "X" on red background (GenBank accession number XP_017505753.1). This theoretical amino acid sequence was aligned to that of human ZBP1. Identical amino acid residues are indicated by blue fonts. Red dashes indicate the absence of the carboxy-terminal protein segment in this prediction. Numbers indicate amino acid positions. Note that additional deleterious mutations that have not been marked by the automatic gene prediction algorithm, are present in the pseudogenes.
