# Supplementary material for: Pangolins Lack IFIH1/MDA5, a Cytoplasmic RNA Sensor That Initiates Innate Immune Defense Upon Coronavirus Infection
Source: Front Immunol. 2020 May 8;11:939. doi: 10.3389/fimmu.2020.00939 (PMC7225364; doi:10.3389/fimmu.2020.00939)
Supplement: Supplementary file 3 [file Image_2.pdf]

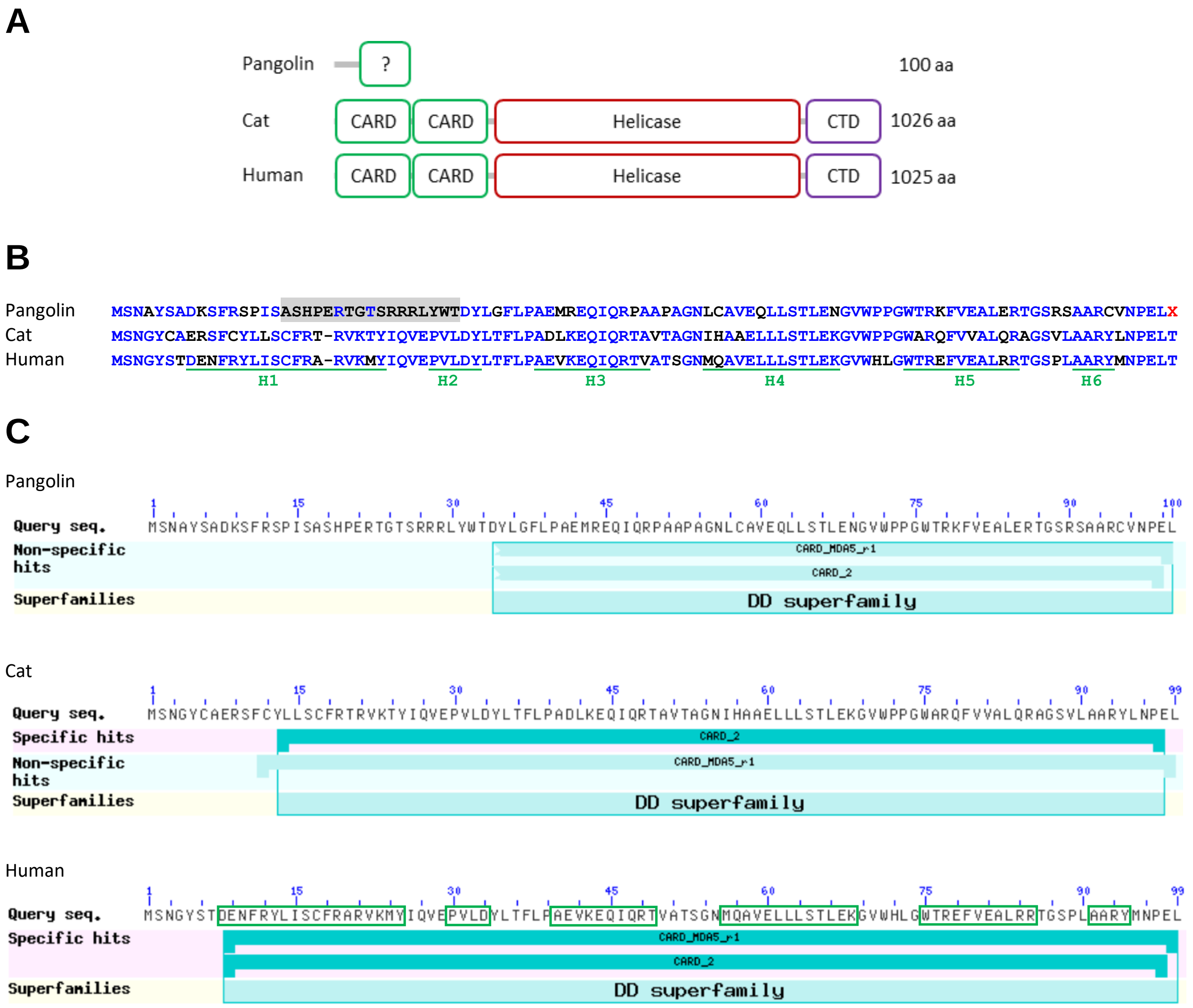

Supplementary Figure S2. Comparison of domain predictions for IFIH1 of cat and human and the *in silico* translation product of *IFIH1* of the Malayan pangolin. (A) Schematic overview of the domain organization of the *in silico* translation product of *IFIH1* of the pangolin (*M. javanica*), in comparison to cat and human IFIH1. Caspase recruitment domains (CARDs), helicase domain and carboxy-terminal domain (CTD) are indicated. A question mark indicates an unknown confirmation of the IFIH1 translation product of the pangolin. The number of amino acids (aa) of the proteins is indicated on the right. (B) Amino acid sequence alignment of the *in silico* translation product of pangolin *IFIH1* and the first CARD of cat and human IFIH1. Amino acid residues identical in 2 or 3 sequences are shown with blue fonts. Grey shading indicates a segment of the pangolin sequence that is encoded by a gene segment flanked by frame-shift mutations (Suppl. Fig. S1A). The red “X” indicates the end of the translation product due to the presence of an in-frame stop codon. The residues that form the 6 helices (H1-H6) of the CARD, according to Potter et al. *BMC Struct Biol.* 2008;8:11, are underlined in the human sequence. (C) Structure prediction of the sequences shown in panel B using the conserved domain search tool of the National Center for Biotechnology Information (NCBI) (Marchler-Bauer A, Bryant SH. *Nucleic Acids Res.* 2004;32:327-331). Alpha-helices corresponding to the prediction in panel B are manually indicated by green boxes. CARD_MDA5_r1, caspase activation and recruitment domain found in MDA5 first repeat; DD, death domain.
