# Supplementary material for: Pangolins Lack IFIH1/MDA5, a Cytoplasmic RNA Sensor That Initiates Innate Immune Defense Upon Coronavirus Infection
Source: Front Immunol. 2020 May 8;11:939. doi: 10.3389/fimmu.2020.00939 (PMC7225364; doi:10.3389/fimmu.2020.00939)
Supplement: Supplementary file 4 [file Image_3.pdf]

A

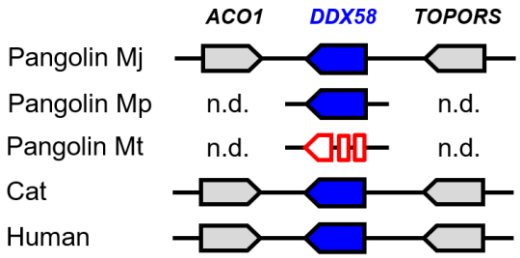

B

```
Pangolin Mj                       G  C  G  K  T  F  V  S  L  L  I  C  E  H  H  L  K  K  F  P  E  G  Q  K
Pangolin Mj   TTTTCTTTATATGTTAAAAGGTTGTGGAAAAACCTTTGTTTCACTTCTTATATGTGAACATCATCTTAAAAAATTTCCAGAAGGACAAAA
Pangolin Mp   TTTTCTTTACATGTTAAAAGGTTGTGGAAAAACCTTTGTTTCACTTCTTATATGTGAACATCATCTTAAAAAATTTCCAGAAGGACAAAA
Pangolin Mt   TTTTCTTTATATGTTAAAAGGTTGTGGAAAAACCTTTGTTTCACTTCTTATATGTGAACATCATCTTTAAAAAATTTCCAGAAGGACAAAA
Cat           TTTTCTTTTTATGTTAAAAGGTTGTGGAAAAACCTTTGTTTCACTTCTTATATGTGAGCATCATCTTAAAAATTTTCCGCAAGGACAAAA
Human         TTTTCCTTACATGTTAAAAGGTTGTGGAAAAACCTTTGTTTCACTGCTTATATGTGAACATCATCTTAAAAAATTCCCACAAGGACAAAA
Human                             G  C  G  K  T  F  V  S  L  L  I  C  E  H  H  L  K  K  F  P  Q  G  Q  K

Pangolin Mj     G  K  V  V  F  F   A  I  Q  L  P  V  Y  E  Q  Q  K  S  V  F  S  Q  Y  F  E  R  L  G
Pangolin Mj   GGGGAAGGTTGTTTTTTTT-GCTATTCAACTCCCAGTATATGAACAGCAGAAATCCGTGTTCTCACAATATTTTGAAAGACTTGGGTAGG
Pangolin Mp   GGGGAAGGTTGTTTTTTTT-GCTATTCAACTCCCAGTATATGAACAGCAGAAATCCGTGTTCTCACAATATTTTGAAAGACTTGGGTAGG
Pangolin Mt   GGGGAAGGCCATTTTTTTTTTGCTATTCAACTCCCAGTATATGAACAGCAGAAATCCGTGTTCTCACAATATTTTGAAAGACGTGGATATG
Cat           GGGGAAGGTTGTCTTTTTT-GCTGTTCAACTCCCAGTGTATGAACAGCAGAAATCTGTGTTCTCAAACTATTTTGAAAGACTTGGGTAGG
Human         GGGGAAAGTTGTCTTTTTT-GCGAATCAGATCCCAGTGTATGAACAGCAGAAATCTGTATTCTCAAAATACTTTGAAAGACATGGGTAGG
Human           G  K  V  V  F  F   A  N  Q  I  P  V  Y  E  Q  Q  K  S  V  F  S  K  Y  F  E  R  H  G
```

Supplementary Figure S3. *DDX58*, encoding the RNA sensor RIG-I, is a pseudogene in the tree pangolin. (A) Gene locus of *DDX58* in pangolins, cat, and human. Genes are represented by arrows pointing in the direction of transcription. The genes flanking *DDX58* could be investigated only in species for which annotated genome sequence assemblies were available, namely the Malayan pangolin (Mj), cat, and human. (B) The nucleotide sequences of exon 7 of *DDX58* of pangolins, cat and human were aligned. The coding sequences of this exon of the Malayan pangolin and of human were translated and the amino acid sequences are shown above and below the nucleotide sequences. An in-frame stop codon and a frameshift mutation in the *DDX58* gene of the tree pangolin (Mt) are highlighted by red shading. Nucleotides conserved in more than 50% of the sequences are indicated by blue fonts. Nucleotides in the introns flanking exon 7 are shown with grey shading. Note that the splice donor site after exon 7 of the tree pangolin is mutated from GT to AT and also other exons of the *DDX58* gene contain inactivating mutations in the tree pangolin. Nucleotide sequence accession numbers (GenBank): Human (NC_000009.12, nucl. 32488729-32488907, compl.), cat (NC_018735.3, nucl. 56896997-56897175, compl.), Malayan pangolin (NW_016527871.1, nucl. 151622-151800, compl.), Chinese pangolin (JPTV01088258, nucl. 5278-5456, compl.), Tree pangolin (SOZM010007722, nucl. 59731-59910, compl.). Abbreviations: Mj, *Manis javanica*; Mp, *Manis pentadactyla*; Mt, *Manis tricuspis*; compl., complementary; n.d., not determined; nucl., nucleotide numbers.
